# Burnout and its’ associated factors among health workers: A hospital-based cross-sectional study from Pokhara, Nepal

**DOI:** 10.1371/journal.pone.0360067

**Published:** 2026-10-08

**Authors:** Dikshya Adhikari, Hari Prasad Kaphle, Ramesh Ojha, Chiranjivi Adhikari

**Affiliations:** School of Health and Allied Sciences, Pokhara University, Pokhara, Nepal; Chengdu Fifth People's Hospital, CHINA

## Abstract

**Background:**

Health workers’ burnout compromises patient safety and satisfaction, in addition to their own health. Over the last three decades or more, burnout syndrome has been getting more attention. High levels of burnout have been reported by health workers globally, and Nepal is no exception, including those mainly in hospital settings. Despite this, burnout issues have not been given enough attention in the literature. Hence, this study aims to assess the prevalence of burnout among health workers in hospital settings in Pokhara and to assess its’ associated factors (personal, job-related, and work-life balance).

**Materials and methods:**

A hospital-based cross-sectional observational study was carried out among 242 health workers from selected hospitals of Pokhara, Kaski, over a period of one month from Sep to Oct, 2024. Data were collected using a self-administered questionnaire to assess the prevalence of burnout and its’ associated factors. The Oldenburg Burnout Inventory scale was used for assessing the prevalence of burnout. Frequency, percentage, and median were calculated to describe the sample characteristics, whereas, chi-square tests and logistic regression were used to determine the factors associated with burnout.

**Results:**

The prevalence of burnout was 61.6%. When subscales were evaluated separately, slightly more than and nearly three-fourths were found to have both high disengagement (76.4%) and high exhaustion (71.9%). Multivariate logistic regression showed that the insufficient sleeping hours at night (aOR: 2.292, 95% CI: 1.193–4.404), poor job satisfaction (aOR: 3.391, 95% CI: 1.286–8.943), and poor work-life balance (aOR: 5.187, 95% CI: 2.600–10.350) were associated with burnout status.

**Conclusions:**

Nearly two-thirds of the hospital health workers experienced burnout in Pokhara. Insufficient sleeping duration at night, poor job satisfaction, and poor work-life balance determined the burnout status of health workers. Hospital administrators, policymakers, and health system leaders should prioritize interventions targeting burnout among health workers, which protect work-life balance and improve job satisfaction.

## Introduction

Health workforce is one of the components of World Health Organizations’ (WHO’s) six building blocks of health system [1]. The burden associated with work has grown during the last few decades. It can lower employees’ self-efficacy and negatively affect their health, ultimately causing professional burnout [2]. Over the course of the last thirty years, researchers, practitioners, and members of the public worldwide have experienced burnout syndrome [3]. High levels of burnout were reported by health workers globally, and their well-being directly affects the quality of care they provide [4].

Burnout has been conceptualized as a syndrome that occurs as a response to chronic workplace stress [5]. The Oldenburg Burnout Inventory scale operationalizes disengagement and exhaustion as the syndrome’s two main dimensions [6]. Disengagement refers to the process of withdrawing from an activity or situation. Exhaustion refers to a state of extreme physical or mental tiredness [7]. Burnout has serious and complex effects on healthcare professionals. The mental and physical health of healthcare professionals is adversely affected by burnout, which results in increased stress, fatigue, and emotional exhaustion [8].

According to the Centers for Disease Control and Prevention (CDC), compared to 32% in 2018, 46% of healthcare professionals said they experienced burnout frequently or very frequently in 2022 [9] A meta-analysis conducted on 2024 from 92 studies found that burnout rates ranging from 38% in high-income countries to 65% in low- and middle-income countries, emphasizing systemic disparities in healthcare resources and workforce pressures [10]. In South Asia, a systematic review done on 2023 highlighted burnout rates range from 52–82% among nurses and doctors, due to lack of sufficient staffs, long shifts, and limited institutional support [11]. In North and South America, the frequency of burnout syndrome among healthcare personnel ranged from 1.3% to 73.5%, whereas in Asia, it varied from 5.2% to 82.1%, according to a study conducted between 2018 and 2022 [3]. In Nepal, a moderate level of burnout (89.5%) has been reported among healthcare personnel [12]. 70.6% of healthcare personnel experienced burnout, according to a web-based cross-sectional study done in Dhulikhel Hospital, Kathmandu University, Nepal, during the Covid-19 pandemic [13]. The primary causes of burnout among healthcare professionals were dealing with patient parties, administrative tasks, and time constraints [12]. An analytical study carried by Shrestha et al. In 2021, revealed that among doctors, 89% of doctors had experienced moderate burnout, and among nurses, 92.2% had moderate burnout. The study also identified that time pressure (22.2%), administrative work (20.1%), and dealing with patients’ relatives (13.5%) are the main sources of burnout [12].

Numerous studies have identified workload, sleep deprivation, a stressful work environment, exposure to workplace violence, and many other personal and job-related factors as common factors associated with burnout [8,14]. Since the majority of attention is focused on the occupational health and safety of health workers resulting from various dangers related to work-related activities, burnout issues among health workers in Nepal have not received enough attention [12]. Achieving universal health coverage depends heavily on human resources for health, which form the backbone of every nation's healthcare system [1]. Because of the demanding nature of their work, health workers are particularly susceptible to burnout, which manifests as a progressive loss of idealism, excitement, or motivation. Addressing burnout through health-promoting strategies such as mindfulness programs, flexible scheduling is essential for ensuring patient safety, maintaining a resilient healthcare workforce, and achieving broader health system goals [15]. Nepal’s National Human Resources for Health (HRH) Strategy 2030 identifies improving health worker retention, performance, and well-being as key national goals to realize Universal Health Coverage. Without addressing burnout, staff turnover will likely rise, productivity will drop, and the already strained health workforce will weaken even more [16].

Although burnout is the most significant problem among health workers at all levels of hospitals nationwide, very limited studies have been conducted on burnout and its associated factors in the health sector. The majority of these studies have focused on hospitals in the Kathmandu Valley or were conducted during the COVID-19 period. Additionally, many studies focus only on doctors or nurses rather than including multiple categories of health workers. Therefore, this cross-sectional study aims to find out the prevalence of burnout and its’ associated factors (personal, job-related, and work-life balance) among health workers in Pokhara, Nepal.

## Materials and methods

### Study design and settings

This study employed a hospital-based cross-sectional observational design conducted over a period of one month from Sep to Oct 2024. The study focused on the hospitals in Pokhara, Nepal. There isa total of 44 hospitals in Pokhara. Among them, 4 are public hospitals and 40 are private hospitals [17]. Pokhara is the capital city of Gandaki Province and the second-largest city in Nepal by population. A large number of health workers are working in hospitals in Pokhara, making it an ideal location to assess the prevalence of burnout and its’ contributing factors among health workers.

### Study participants

The participants in this study consisted of health workers (doctors, nurses, and paramedics) working in the selected hospitals in Pokhara, Nepal. The inclusion criteria required health workers to be working there for more than six months in the different departments, available during the data collection period, and volunteered to participate. Health workers of hospitals in Pokhara who were on vacation, on leave, or refused to participate in the study were excluded. These criteria were established considering that these could interfere with the quality of the information given.

### Sample size determination and sampling procedure

The sample size was calculated by using the finite population correction (Cochrane) formula with a 5% margin of error and a 95% confidence level and taking the prevalence of burnout in Nepal (p)= 70.6% = 0.706 [13] Applying a design effect of 1.2, the final sample size was 242.

Firstly, we selected two public (50% of all public hospitals) and six private hospitals (15% of all private hospitals) from a total of 44 Hospitals (4 public and 40 private) in Pokhara. This ensured proportional representation of both sectors based on their contributions to the total hospital workforce. Health workers were then grouped by job type/nature into 3 strata – doctors, nurses, and paramedics and the sample size for each stratum was allocated proportionate to its size. Finally, individuals were randomly selected within each stratum using a lottery method (Fig 1).

**Fig 1:**
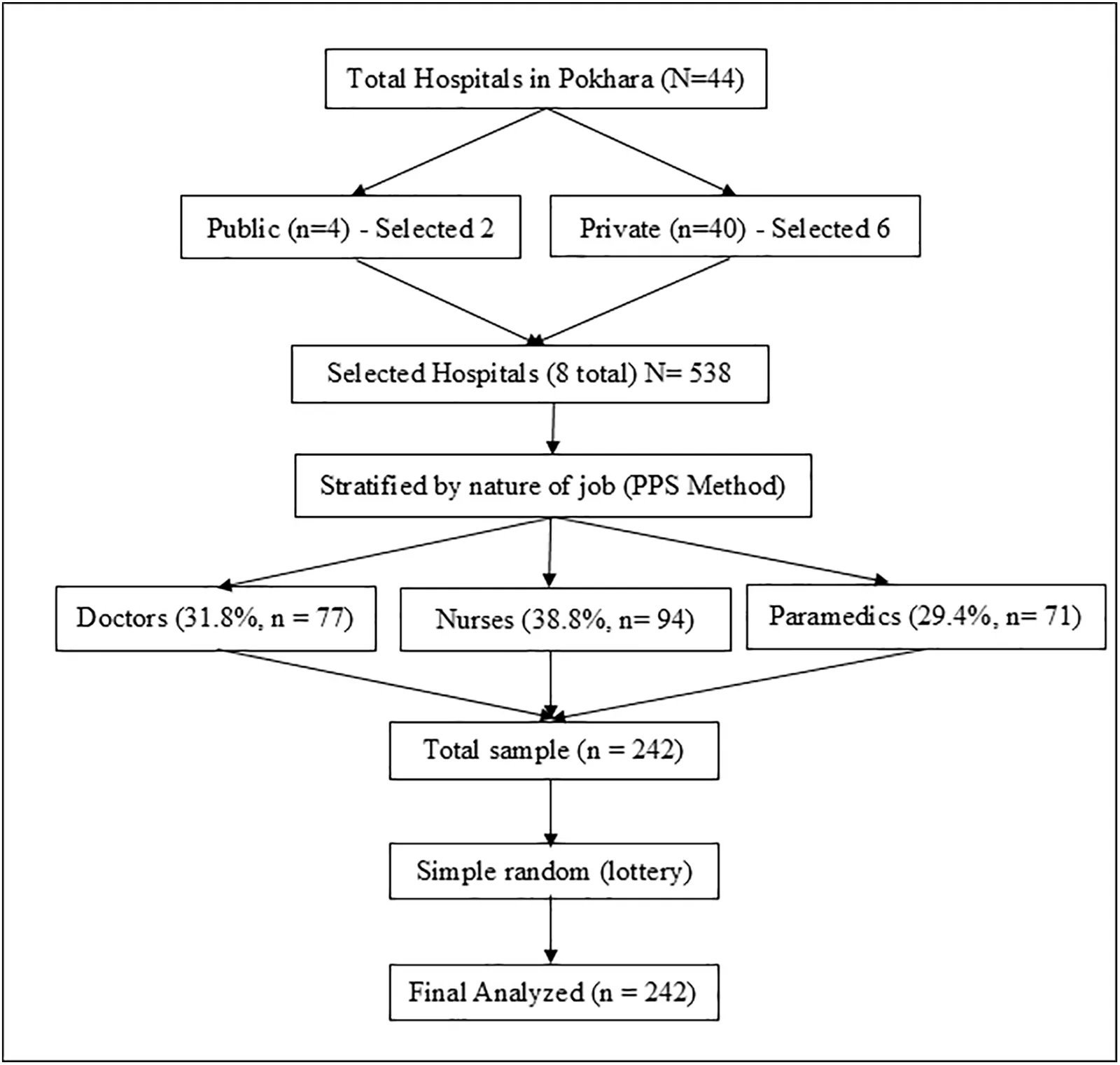
Sampling procedure.

### Data collection tools and procedure

Data were collected through the pre-tested self-administered questionnaire. The questionnaire was structured into 4 sections: personal information, job-related information, work-life balance related information, and burnout related information. Section A consists of personal information, which includes age, sex, professional qualification, marital status, parental status, and sleeping hours at night. Section B consists of job-related information. It includes work duration, working hours, work-shift pattern, workload, social support, job safety and security, and job satisfaction. Tools for these were prepared based on supervisors’ consultation and existing literature.

Job satisfaction was measured on a 4-point Likert scale from very satisfied to very dissatisfied, with responses of very dissatisfied and dissatisfied indicating poor job satisfaction, and satisfied or very satisfied indicating good job satisfaction as per consultation with supervisor. Similarly, Section C consists of work-life balance related information. For its’ measurements, the work-life balance scale developed by Hayman (2005) was used. The original scale consists of 15 questions with 3 dimensions of work-life balance: work interference with personal life (WIPL), personal life interference with work (PLIW), and work/personal life enhancement (WPLE) [18]. A modified questionnaire of Hayman (2005) was used, which demonstrated acceptable internal consistency (Cronbach’s alpha = 0.812) in the present study. Each question’s replies were given a score between 1 and 5 (1 being strongly disagree, 2 disagree, 3 being neutral, 4 being agree, and 5 being strongly agree). The analysis was done by calculating the median score of each category and median was taken as cutoff points to. categorize work-life balance as balanced (good) or imbalanced (poor) [19].

Moreover, section D consists of burnout-related information. The data collection tool was adopted from the; Oldenburg Burnout Inventory Scale for measuring the level of burnout among health workers. This tool was originally developed by Demerouti et al. in 2003. The tool consists of 2 items: exhaustion and disengagement. The participant was asked to indicate how much they agreed or disagreed with a set of sixteen items. The 16 items are divided into two categories: items 2, 4, 5, 8, 10, 12, 14, and 16 are for exhaustion, and items 1, 3, 6, 7, 9, 11, 13, and 15 are for disengagement. The scores for the four negative elements in both sections must be reversed, going from 1 to 4, 2–3, 3–2, and 4–1. Each part’s average was computed separately. The cutoffs for exhaustion and disengagement were determined to be scores more than or equal to 2.25 and greater than or equal to 2.1, respectively. Exhaustion and disengagement were categorized as high and low based on cutoff values. High exhaustion and high disengagement were grouped as burnout, whereas others were not burnout [6,20]. The English translation of the tool was done in 2005, having Cronbach’s alpha for exhaustion at 0.74 and disengagement at 0.76 [21].

Furthermore, an extensive literature review and consultation with the supervisor, and subject expert was done to ensure validity. The reliability of the tools was established by pre-testing the tools among 10% (i.e., 32) of the sample. The questions were read and verified to ensure that they are simple, easy to understand, and relevant to the research interest.

### Operational definitions

1 **Work-life balance:** Work-life balance (WLB) in this study refers particularly having enough time and energy for both job and personal life of health workers without letting one interfere with the other. It is measured by Work-life balance scale developed by Hayman, 2005 consisting of three dimensions: work interference with personal life, personal life interference with work and work personal life enhancement [18]. It is calculated based on the median of each dimension.
2 **Burnout:** Burnout is measured by Oldenburg Burnout Inventory Scale consisting of 16 questions. Burnout often results in disengagement and exhaustion. The subscale disengagement and exhaustion are categorized as high and low based on cutoff value. High disengagement and high exhaustion are categorized as burnout and others as no burnout [6,20].
3 **Perceived workload:** Workload in this study can be defined as having too much work to do in too little time. It is categorized as: Low, Moderate and Heavy.
4 **Perceived social support:** Social support in this study refers to the support and assistance that health professionals give to each other and receive from supervisors. It is categorized as: Not any support at all, Moderate support and Strong support.
5 **Job satisfaction:** Job satisfaction in this study refers particularly to the amount of satisfaction and dissatisfaction that health workers experience in their jobs in hospitals. It is categorized as: Very satisfied, Satisfied, Dissatisfied, and Very dissatisfied.

### Data management and analysis

Epi-Data version-3.1 was used for data entry, and Statistical Package for Social Sciences (SPSS) version-25 was used for data management and analysis. Data cleaning was performed to check for consistencies and values, and analysis was done in three stages. In the 1st stage, descriptive statistics were used to summarize the independent and dependent variables. Continuous variables were presented as median and quartile after checking for normal distribution, whereas categorical variables were presented as frequencies and percentages. The normality of continuous variables was explored visually (histogram) and numerically (Kolmogorov-Smirnov tests and Shapiro-Wilk test). Similarly, the chi-square test (p-value <0.05 at 95% level of confidence) was applied to test the association between dependent and independent variables.

Similarly, binary logistic regression analysis was applied to check the association between dependent and independent variables in which all variables that were significant in the Chi-square test were included. Crude and adjusted odds ratios with 95% confidence intervals were calculated, and p-values < 0.05 were used to determine whether a variable was statistically significant.

Furthermore, multicollinearity among independent variables was measured by using the tolerance and Variance Inflation Factor (VIF). Maximum tolerance of 0.951 was less than 1, and maximum VIF of 1.278 was less than 5, so there were no threats of multicollinearity. Parameters such as Cox and Snell Pseudo R-squares, Nagelkerke, and the Hosmer and Lemeshow test were performed to determine the goodness of fit with the model and the variance of independent variables that explained burnout status. Further, we carried out a sensitivity analysis to examine the margin of errors (MoEs) with hypothetical simulations of 10% and 20% attritions of sample. The MoEs (at 95% confidence limits) increased from 4.68% to 5.06% and 5.51% at 10% and 20% attritions, respectively (S1 Table). This showed negligible increments in MoE even at severe attritions.

### Ethical approval

Ethical approval was taken from the Institutional Review Committee (IRC) of Pokhara University (Reference Number: 95/2081/82, approval date: August 30, 2024). Similarly, formal permission was obtained from selected hospitals of Pokhara for data collection. Written informed consent was taken from each participant, and they were informed of their right to withdraw from the study at any time without reason. Data obtained from the study participants were kept confidential and not used for any other purpose than research.

## Results

### Personal information of health workers

The median age of the health workers was 26 years (IQR: 5, min. 18 and max. 55), where nearly half (45.9%) of the health workers had their age between 26–30 years. More than two-thirds (70.2%) of the health workers were female. Similarly, slightly less than half (43.4%) of the health workers had completed a bachelor’s level of education, followed by certificate level of education (36.8%). Among health workers, nurses (38.8%) were slightly more than doctors (31.8%) and Paramedics (29.3%). More than half (58.3%) of the health workers were unmarried. Nearly three-fourths (73.1%) of the health workers didn’t have children. More than half (53.7%) of the health workers slept less than 8 hours at night (Table 1).

**Table 1:** Personal information of health workers.

Variables (n = 242)	Frequency (n)	Percentage (%)
**Age of health workers [Md = 26, IQR = 5, Min = 18, Max = 55]**		
≤20 years	11	4.5
21-25 years	75	31.0
26-30 years	111	45.9
31-35 years	24	9.9
<35 years	21	8.7
**Sex of health workers**		
Male	72	29.8
Female	170	70.2
**Level of education**		
Technical SLC	26	10.7
Certificate level	89	36.8
Bachelor	105	43.4
Masters	22	9.1
**Category of health worker**		
Doctor	77	31.8
Nurse	94	38.8
Paramedics	71	29.3
**Marital status**		
Unmarried	141	58.3
Married	101	41.7
**Number of children**		
0	177	73.1
1	43	17.8
≥2	22	9.1
**Sleeping hours at night [Md = 7, IQR = 4, Min = 6, Max = 10]**		
<8 Hrs	130	53.7
≥8 Hrs	112	46.3

### Job related information of health workers

Regarding total work experience of health workers, more than two-thirds (69.8%) of them had up to 5 years of work experience, followed by 6–10 years (19.8%) in Hospital. Similarly, nearly one-fourth (28.5%) of them had 2 or more than 2 years of work experience in same Hospital. The median working hours of health workers were 6 hours and 48 hours in a day and week, respectively, where the majority of health workers worked up to 8 hours per day (86.0%) and up to 48 hours in a week (83.9%). Nearly three-fourths (71.5%) of the health workers were working both shifts (day and night) in a hospital. The median breaktime in duty hours was 30 minutes, where more than half (51.2%) of health workers had had more or equal to 30 minutes break in duty hours. Similarly, most (85.5%) of the health workers perceived their workload as moderate. Most (83.5%) of the health workers hadn’t been threatened, assaulted, or bullied in Hospital. Likewise, nearly three-fourths (71.1%) of the health workers had perceived moderate safety in their workplace. More than one-third (35.5%) of them had perceived strong social support and more than three-fourths (70.2%) were satisfied in their job. Similarly, most (84.7%) of them had interest in their own profession (Table 2).

**Table 2:** Job related information of health workers.

Variables (n = 242)	Frequency (n)	Percentage (%)
**Total work experience [Md = 3 yrs, IQR = 5 yrs, Min = 7 m, Max = 30 yrs]**		
≤ 5 years	169	69.8
6-10 years	48	19.8
11-15 years	13	5.4
>15 years	12	5.0
**Total work experience in same hospital [Md = 16 m, Min = 7 m, Max = 22 yrs]**		
< 2 years	173	71.5
≥ 2 years	69	28.5
**Working hours in a day [Md = 6, IQR = 2, Min = 6, Max = 24]**		
≤ 8 Hrs/day	208	86.0
> 8 Hrs/day	34	14.0
**Working hours in a week [Md = 48, IQR = 6, Min = 18, Max = 72]**		
≤ 48 Hrs/week	203	83.9
> 48 Hrs/week	39	16.1
**Work-shift pattern**		
Day shift	69	28.5
Both shift	173	71.5
**Breaktime in duty hours [Md = 30, IQR = 20, Min = 0, Max = 30]**		
No break	55	22.7
<30 minutes	63	26.0
≥30 minutes	124	51.2
**Perceived workload**		
Moderate	207	85.5
Heavy	35	14.5
**Experience of being threatened, assaulted, or bullied**		
Yes	40	16.5
No	202	83.5
**Perceived workplace safety**		
Low	27	11.2
Moderate	172	71.1
High	43	17.8
**Perceived social support**		
Not any support at all	15	6.2
Moderate support	142	58.7
Strong support	85	35.5
**Job satisfaction**		
Very satisfied	21	8.7
Satisfied	170	70.2
Dissatisfied	45	18.6
Very dissatisfied	6	2.5
**Interest in own profession**		
Yes	205	84.7
No	37	15.3

### Work-life balance of hospital health workers

Regarding work-life balance information, more than half (51.7%) of the health workers had balanced work-life. When the components of work-life balance were evaluated separately, less than half (45.9%) of the health workers reported that work interfered with personal life. Similarly, more than one-third (38.4%) of them reported that personal life interfered with work. Slightly less than half (48.3%) of the health workers reported that work didn’t enhance personal life (Table 3).

**Table 3:** Work-life balance related information of health workers.

Characteristics	Frequency (%)	
	Yes	No
Work interference with personal life	111 (45.9)	131 (54.1)
Personal life interference with work	93 (38.4)	149 (61.6)
Poor work/personal life enhancement	117 (48.3)	125 (51.7)
Work-life balance	125 (51.7)	117 (48.3)

### Burnout related information of health workers

Nearly two-thirds (61.6%) of the health workers had experienced burnout (Fig 2). More than three-fourths (76.4%) of health workers had a high level of disengagement, and less than three-fourths (71.9%) of them had a high level of exhaustion (Fig 3).

**Fig 2:**
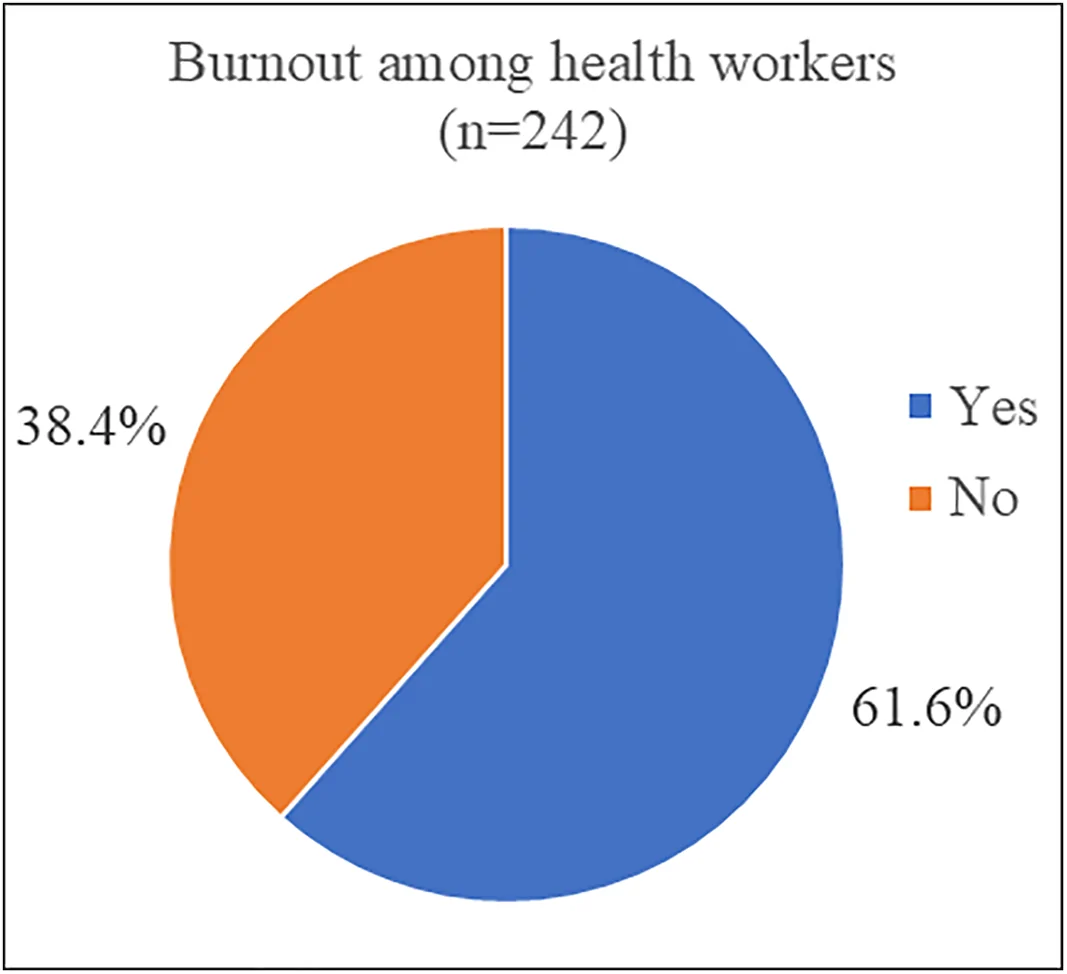
Burnout among health workers.

**Fig 3:**
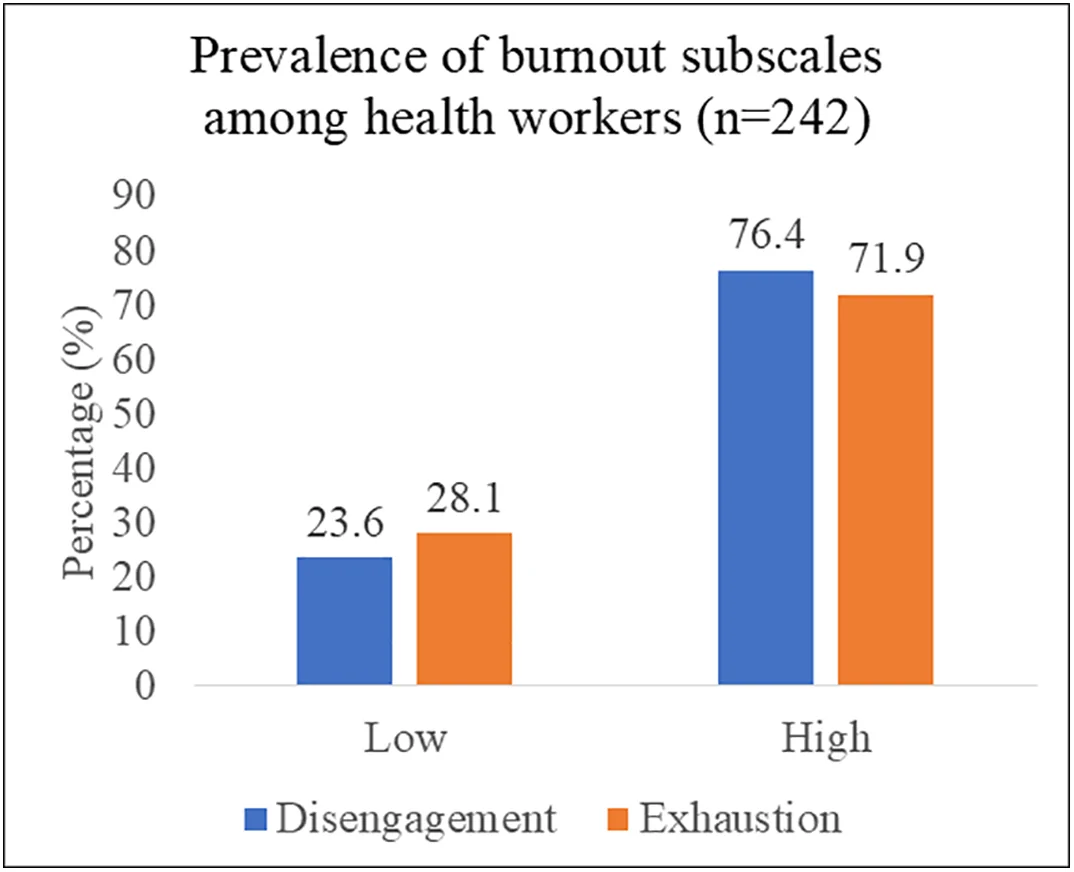
Prevalence of burnout subscales among health workers.

### Factors associated with burnout among health workers from multi-variate logistic regression

The odds of having burnout were higher among health workers who slept for < 8 hours at night (p: 0.013, aOR: 2.292, 95% CI: 1.193–4.404) compared to those who slept for ≥ 8 hours at night. Likewise, health workers who were satisfied in their job (p: 0.014, aOR: 3.391, 95% CI: 1.286–8.943) were 3 times more likely to have burnout compared to those who were dissatisfied. Similarly, the odds of having imbalanced work-life balance among health workers (p: 0.000, aOR: 5.187, 95% CI: 2.600–10.350) were higher compared to those with a balanced work-life balance (Table 4).

**Table 4:** Factors associated with burnout among health workers from multi-variate logistic regression.

Variables (n=242)	cOR		aOR	
	p-value	95% CI	p-value	95% CI
**Sex of health workers**				
Male	-	Ref.	-	Ref.
Female	0.017*	1.982 (1.131 -3.474) *	0.149	1.859 (0.801-4.316)
**Education of health workers**				
Technical SLC	-	Ref.	-	Ref.
Certificate level	0.947	1.031(0.425-2.497)	0.477	1.466(0.511-4.206)
Bachelors’	0.900	1.057 (0.443-2.523)	0.932	1.950(0.293-3.084)
Masters’	0.018*	7.333 (1.410 – 38.134) *	0.054	7.364(0.963-56.333)
**Category of health workers**				
Doctor	0.010*	2.403 (1.229-4.698) *	0.505	1.489(0.462-4.798)
Nurse	0.021*	2.108 (1.121-3.965) *	0.384	1.446(0.631-3.314)
Paramedics	-	Ref.	-	Ref.
**Marital status**				
Unmarried	-	Ref.	-	Ref.
Married	0.037*	1.772 (1.035-3.035) *	0.178	1.645(0.798-3.393)
**Sleeping hours at night**				
< 8 hrs/day	0.027*	2.706(1.587-4.614) *	0.013**	2.292(1.193-4.404)
≥ 8 hrs/day	-	Ref.	-	Ref.
**Working hours in a day**				
≤ 8 hrs/day	-	Ref.	-	Ref.
> 8 hrs/day	0.025*	2.719 (1.132-6.528) *	0.060	2.671(0.960-7.431)
**Breaktime during duty hours**				
Yes	-	Ref.	-	Ref.
No	0.012*	2.416 (1.216-4.798) *	0.143	1.845(0.813-4.187)
**Perceived workload**				
Moderate	-	Ref.	-	Ref.
Heavy	0.019*	2.843 (1.187-6.807) *	0.421	1.534(0.541-4.352)
**Experienced of being threatened, assaulted or bullied**				
Yes	0.027*	2.452 (1.109-5.420) *	0.302	1.691(0.624-4.585)
No	-	Ref.	-	Ref.
**Perceived safety**				
Low	0.022*	3.299 (1.184-9.188) *	0.153	2.558(0.706-9.268)
Moderate	0.288	2.593 (1.311-5.129)	0.065	2.287(0.949-5.510)
High	-	Ref.	-	Ref.
**Job satisfaction**				
Satisfied	-	Ref.	-	Ref.
Dissatisfied	0.001*	3.664 (1.689-7.948) *	0.014**	3.391(1.286-8.943)
**Interest in own profession**				
Yes	-	Ref.	-	Ref.
No	**0.011**	3.097 (1.300-7.380) *	0.056	2.967(0.970-9.072)
**Work-life balance**				
Yes	-	Ref.	-	Ref.
No	0.000*	2.947(1.712-5.073) *	0.000**	5.187(2.600-10.350)

cOR = crude odds ratio; aOR = adjusted odds ratio; CI = confidence interval

* Significant at p < 0.05 from bivariable binary logistic analysis

** Significant at p < 0.05 from multivariable binary logistic analysis

Cox & Snell R Square: 0.301

Nagelkerke R Square: 0.408

Hosmer and Lemeshow test (p-value): 0.278

## Discussion

This study assessed burnout and its’ associated factors among health workers in hospitals of Pokhara, Nepal. The overall prevalence of burnout in this study’s findings is 61.6%.However, a systematic review conducted before the COVID-19 pandemic reported that burnout was prevalent among physicians, nurses, and other healthcare providers in sub-Saharan Africa, with prevalence estimates ranging from 40% to 80% [22]. This findings is consistent with finding of the study conducted at Dhulikhel Hospital in Nepal in 2023, where the prevalence of burnout for healthcare workers was 70.6% [13]. Similarly, 62% of burnout was found among healthcare workers in the study performed at a national referral hospital in Tanzania in 2021 [23]. The high rates of burnout in this study could be due to work-related stress, poor infrastructure and lack of suitable accommodation. Moreover, it might be due to country’s weak healthcare system and economic meltdown.

In contrast, the findings of this study were higher than the study conducted among healthcare workers in COVID-19 isolation centers in Khartoum, Sudan, in 2022, where burnout prevalence was 45.7% [7]. Similarly, 31.4% of high burnout was found among public doctors in Hong Kong in 2012 [24]. Likewise, the overall prevalence of burnout syndrome among healthcare professionals was 15.1% (95% CI 10.8–19.9) in the study conducted at Dessie Comprehensive Specialized Hospital, Ethiopia, in 2021 [25]. The possible reasons for variation might be due to the respondents’ socio-demographic factors, sample size, study settings and use of different screening tools.

When subscales were evaluated separately, 76.4% met high disengagement and 71.9% met high exhaustion in this study. In contrast, the findings of this study were lower than the study conducted among public health workers in Canada in 2023, where 87.7% met the criteria for disengagement and 85.2% met the criteria for exhaustion [26]. The possible reasons for variation might be due to the immense burden associated with managing long term response to the COVID-19 pandemic professionally and personally and work-related stress among public health workforce. The findings of this study indicated that a high prevalence of burnout was found among health workers working in different hospitals. The finding of this study highlights the urgent need for hospitals and policymakers in Pokhara as well as Nepal to implement strategies such as flexible shift scheduling, mental health support, and initiatives to enhance job satisfaction and work-life balance.

In this study, sleeping <8 hours at night had higher odds (p = 0.013, aOR: 2.292, 95% CI: 1.193–4.404) of having burnout compared to those who slept for more or equal to 8 hours. The strongest correlation was found between workplace burnout and lack of sleep in the study conducted in Taiwan in 2019 [27]. Likewise, another study conducted among healthcare workers’ at a tertiary teaching hospital in Tanzania in 2021 showed that the participants who slept more hours at night had lower odds of having burnout compared with those who slept for less than 4 hours per night (p < 0.001) [23]. Similarly, the participants who reported <7 hrs of sleep had (aOR: 10.86, 95% CI: 6.48–15.24) more burnout points compared to those who slept ≥7 hrs in the study conducted in HCPs in Peru in 2023 [28]. Also, the study conducted in Sweden in 2012 showed that too little sleep (<6 Hrs) was the main risk factor for clinical burnout after adjustment of covariates [29]. These findings could be due to long working hours, rotating shifts, and the physical and emotional demands inherent to working in the hospital. This indicated that sleeping hours at night also contributed to the prevalence of burnout. Ensuring health workers get at least 8 hours of sleep is crucial, as those sleeping less face a much higher risk of burnout. Hospitals should adjust shift schedules and encourage healthy sleep habits to protect staff well-being, reduce exhaustion, and maintain high-quality patient care.

Health workers who were satisfied in their job (p: 0.014, aOR: 3.391, 95% CI: 1.286–8.943) were 3 times more likely to have burnout compared to those who were dissatisfied in this study. Similar to this study, the odds of burnout syndrome among HCPs who were not satisfied with their job were about 4.17 times higher as compared with the satisfaction with their job (AOR = 4.17:95% CI 1.46–11.90) conducted in the study among HCPs working in Dessie Comprehensive Specialized Hospital, Dessie, Ethiopia in 2021 [25]. Likewise, a systematic review done in 2017 in Arab countries among healthcare providers’ showed that low job satisfaction was associated with a high level of burnout in many studies [30]. The Pearson correlation coefficient result done among public doctors in Hong Kong in 2012 showed that burnout dimensions of Emotional Exhaustion (EE) (r = −0.583, p < 0.001), Depersonalization (DP) (r = −0.578, p < 0.001) and Personal Accomplishment (PA) (r = 0.610,p < 0.001) were inversely related to job satisfaction in doctors [24]. Findings in our study are similar with all other findings, as job satisfaction is measured using simple 4-point Likert scale, which is the simplest way to monitor workforce morale and risk of burnout. This indicated that low in job satisfaction could serve as a less effective way to prevent burnout among health workers. To address this factor, hospitals should focus on improving workplace conditions, recognizing staff contributions, and providing opportunities for professional growth and support, which can reduce burnout, and ultimately enhance patient care quality.

The findings in this study showed that the odds of having an imbalanced work-life among health workers (p: 0.000, aOR: 5.187, 95% CI: 2.600–10.350) were higher compared to those with having work-life balance. This means imbalanced work-life is strongly associated with having burnout. Similar to this study, work-life balance issues contributed significantly to burnout levels (aOR: 0.44, at p ≤ 0.001) in the study conducted among general practitioners conducted in Germany in 2019 [31]. Likewise, job burnout is negatively impacted by work-life balance, according to a study conducted among nurses in Indonesia in 2023 [32]. Another study conducted among emergency healthcare professionals during the COVID-19 pandemic in England in 2022 revealed that there was a statistically significant negative correlation between their total WLBS and MBI scores (*P*  <  0.05). Participants’ work-life balance scores explained 28.5% of the variance in burnout levels (*R* ^2^  =  0.285) [33]. The possible reasons for this findings could be organizational issues, role conflicts, lack of prioritization on the personal health of health workers. This indicated that paying attention to one’s nonprofessional life has a protective role against burnout. The findings of this study showed that imbalance work-life balance greatly increases the risk of burnout among health workers. So, hospitals should support flexible scheduling, encourage time off, and create policies that help staff better manage their personal and professional responsibilities.

Burnout among healthcare professionals leads to emotional exhaustion and disengagement, which directly reduced patient safety, lower patient satisfaction, and an increased risk of adverse events. Over time, high burnout rates contribute to staff turnover and shortages, further straining the healthcare system and reducing the overall quality and continuity of care provided to patients. Hence, addressing burnout is essential for maintaining both patient safety and high standards of care delivery in the hospitals.

This study also has some limitations. The use of self-administered questionnaires may have led to recall or social desirability bias to some extent. Moreover, since only 18.18% of hospitals in Pokhara Metropolitan City were included, the study may be subject to institutional selection bias. Therefore, the findings should be interpreted cautiously when generalizing to other healthcare facilities or to a wider population. Although the modified Hayman work-life balance scale demonstrated acceptable internal consistency in our study (Cronbach’s α = 0.812), factor analysis was not conducted to confirm its underlying dimensional structure in our study population. Finally, null distribution in low workload and non-significance of workload in bivariate analysis doubt its’ normality and so cautiously interpreted.

## Conclusion

It is concluded that nearly two-thirds of the health workers working in hospitals in Pokhara experienced burnout with high level of exhaustion and disengagement. Sleeping less than eight hours at night, poor job satisfaction, and poor work-life balance were found as associated factors for burnout among health workers. This study’s findings align with Nepal’s National Human Resources for Health Strategy 2030 and WHO guidelines, highlighting the need for integrated policy actions to prevent burnout and improve health system functioning. Hospital administrators, policymakers, and health system leaders should prioritize interventions that protect work-life balance and improve job satisfaction to reduce burnout among health workers. Further, longitudinal studies and intervention-based research with larger sample sizes sufficing for each category of health workers are recommended to better understand causal relationships and to identify effective strategies for preventing and reducing burnout among health workers.

## Supporting information

S1 Table Sensitivity analysis of sample. (DOCX)
