# Supplementary material for: Burnout and its’ associated factors among health workers: A hospital-based cross-sectional study from Pokhara, Nepal
Source: PLoS One. 2026 Oct 8;21(10):e0360067. doi: 10.1371/journal.pone.0360067 (PMC13648992; doi:10.1371/journal.pone.0360067)
Supplement: Supplementary file 1 [file pone.0360067.s001.docx]

S1. Table 1. Sensitivity analysis of sample simulation of 10-20% attrition

| Scenario | Attrition % | Effective Sample Size (n) | Population Coverage (%) | Margin of Error (at 95% CL) |
| --- | --- | --- | --- | --- |
| Analyzed sample (baseline) | 0 | 242 | 44.98 | 4.68 % |
| Simulated (attrition 1) | 10 | 218 | 40.52 | 5.12 % |
| Simulated (attrition 2) | 20 | 194 | 36.06 | 5.63 % |
